# Self-microemulsifying drug delivery system and nanoemulsion for enhancing aqueous miscibility of *Alpinia galanga* oil

**DOI:** 10.1371/journal.pone.0188848

**Published:** 2017-11-30

**Authors:** Nattakanwadee Khumpirapang, Surachai Pikulkaew, Anette Müllertz, Thomas Rades, Siriporn Okonogi

**Affiliations:** 1 Interdisciplinary Program in Nanoscience and Nanotechnology, the Graduate School, Chiang Mai University, Chiang Mai, Thailand; 2 Department of Food Animal Clinic, Faculty of Veterinary Medicine, Chiang Mai University, Chiang Mai, Thailand; 3 Department of Pharmacy, Faculty of Health and Medical Sciences, University of Copenhagen, Copenhagen, Denmark; 4 Department of Pharmaceutical Sciences, Faculty of Pharmacy, Chiang Mai University, Chiang Mai, Thailand; Aristotle University of Thessaloniki, GREECE

## Abstract

*Alpinia galanga* oil (AGO) possesses various activities but low aqueous solubility limits its application particularly in aquatic animals. AGO has powerful activity on fish anesthesia. Ethanol used for enhancing water miscible of AGO always shows severe side effects on fish. The present study explores the development of self-microemulsifying drug delivery systems (SMEDDS) and nanoemulsions (NE) to deliver AGO for fish anesthesia with less or no alcohol. Pseudoternary phase diagrams were constructed to identify the best SMEDDS-AGO formulation, whereas NE-AGO were developed by means of high-energy emulsification. The mean droplet size of the best SMEDDS-AGO was 82 ± 0.5 nm whereas that of NE-AGO was 48 ± 1.6 nm. The anesthetic effect of the developed SMEDDS-AGO and NE-AGO in koi (*Cyprinus carpio*) was evaluated and compared with AGO ethanolic solution (EtOH-AGO). It was found that the time of induction the fish to reach the surgical stage of anesthesia was dose dependent. NE-AGO showed significantly higher activity than SMEDDS-AGO and EtOH-AGO, respectively. EtOH-AGO caused unwanted hyperactivity in the fish. This side effect did not occur in the fish anesthetized with SMEDDS-AGO and NE-AGO. In conclusion, SMEDDS and NE are promising delivery systems for AGO.

## Introduction

*Alpinia galanga* is a plant in family Zingiberaceae. Many parts of *A*. *galanga* such as rhizome, flower, and young leaf are edible and have been used in Asian folk medicinal and food remedies. *A*. *galanga* oil (AGO) has wonderful essence that can enhance the flavor of foods and elevate them to a level of brightness. AGO has been extensively used in traditional treatment of stomach ache and diarrhea [1]. Previous studies reported several biological properties of AGO on anti-bacterial, anti-oxidant, and anti-inflammatory activities [2–5]. Interestingly, we have recently found that AGO has excellent action on fish anesthesia [6]. However, the poor water miscibility of AGO causes limitations in its application, particularly in animals for which water is the essential environment, such as fish.

Absolute ethanol has been used to increase the water miscibility of AGO for fish anesthesia but alcohol causes several side effects in fish, such as hyperactivity, a constellation of congenital and retinal anomalies, and central nervous system (CNS) deficits. Ethanol has also been found to induce stress proteins in zebrafish embryos [7–10]. If we can build up an innovative technology to improve the miscibility of AGO in water and avoid using alcohol, we may solve the two main problems (poor solubility in water of AGO and side effect of alcohol) at the same time. Nanoformulations such as self-microemulsifying drug delivery systems (SMEDDS) and nanoemulsions (NE) have been reported to be able to solubilize and stabilize hydrophobic active ingredients and to enhance the bioavailability of many water insoluble compounds [11–14]. Furthermore, SMEDDS and NE have been shown to enhance the biological activity of natural compounds [15–18].

The objective of the present study was to develop and characterize SMEDDS and NE containing AGO (SMEDDS-AGO and NE-AGO) in order to increase AGO water miscibility and to decrease the use of ethanol in fish anesthesia. The effects of type and amount of surfactant used in the developed SMEDDS-AGO and NE-AGO on the phase behavior of the oil/water systems were investigated. In addition, this study also investigated the anesthetic effect of the developed SMEDDS-AGO and NE-AGO in comparison with AGO ethanolic solution (EtOH-AGO) on fish using koi (*Cyprinus carpio*) as an animal model.

## Materials and methods

### Materials

Fresh rhizomes of *A*. *galanga* were collected in December 2015 from the medicinal garden, Chiang Mai, Thailand. The plant voucher specimen no. 009245 was deposited at the Herbarium, Northern Research Center for Medicinal Plants, Faculty of Pharmacy, Chiang Mai University, Thailand. Polyoxyethylene sorbitan monolaurate (Tween 20) and polyoxyethylene sorbitan monooleate (Tween 80) were from Merck (Hohenbrunn, Germany) while polyoxyethylene octylphenyl ether (Triton X-100) was obtained from Amresco (Cleveland, OH, USA). Absolute ethanol, dichloromethane, and hexane were of analytical grade and were supplied by Merck Millipore (Darmstadt, Germany). Isopropanol was purchased from Labscan Analytical Science (Bangkok, Thailand). Propylene glycol was from Sigma-Aldrich (St. Louis, MO, USA). All other chemicals and solvents were of the highest grade available.

### Extraction and chemical analysis of AGO

Fresh rhizomes of *A*. *galanga* were cut into small pieces and subjected to hydrodistillation for 3 h using a Clevenger apparatus for oil collection. The experiment was done in triplicate. The yield of the oil obtained from each experiment was recorded and the average yield value was calculated. The volatile oil obtained was stored in a refrigerator at –20°C and protected from light until use. AGO was analyzed for its chemical composition using gas chromatography-mass spectrometry (GC-MS). An Agilent 6890 gas chromatograph was coupled to an electron impact (EI, 70 eV) using a Hewlett Packard (HP) mass selective detector (MSD), model HP 5973-MSD and fitted with a fused silica capillary column (HP5-MSI; 30.0 m × 0.25 mm i.d. × 0.25 μm film thickness) (Agilent Technologies Inc, USA). The analytical protocol was standardized from previous studies with some modification [19]. Briefly, 1 μL of AGO 1:100 (v/v) diluted with dichloromethane was injected. Identification of the compounds of interest was performed based on comparing retention times to those of reference spectra obtained from two mass spectral databases (Wiley and the National Institute of Standards and Technology; NIST). The Wiley database is the largest database of its kind and the NIST mass spectral database contains approximately 62,000 mass spectra of an equal number of chemicals [20]. These two mass spectral databases are commonly applied for identifying bioactive compounds in natural plants [21–22]. The percentage of each compound was calculated based on the total area of all peaks obtained from the oil.

### Preparation of SMEDDS-AGO

Pseudoternary phase diagrams of AGO were constructed using an aqueous titration method. To obtain the surfactant mixture (Smix), surfactant (Tween 20, Tween 80, or Triton X-100) and cosurfactant (absolute ethanol, propylene glycol, or isopropanol) were mixed at weight ratios of 1:2, 1:1, and 2:1. Then, AGO and Smix were mixed at weight ratios of 0:1, 1:9, 2:8, 3:7, 4:6, 5:5, 6:4, 7:3, 8:2, 9:1 and 1:0. The resulting mixtures were subsequently titrated with water under moderate agitation at ambient temperature. Clear, monophasic liquid samples were classified as microemulsions (ME). All formulations were prepared in triplicate. The pseudoternary phase diagrams were designed using OriginPro8 for Windows (OriginLab Corporation, USA).

Selected formulations of SMEDDS-AGO were then prepared by mixing AGO and several Smix, based on the pseudoternary phase diagrams. Smix was prepared before mixing with AGO using a vortex mixer to the desired weight ratios before transferring to glass vials for further use.

### Preparation of NE-AGO

Six formulations of NE-AGO (A, B, C, D, E, and F), composed of 20% AGO, were prepared by means of high-energy emulsification using three different surfactants; Tween 20, Tween 80, and Triton X-100. NE-AGO-A and NE-AGO-B contained 5% and 10% Tween 20, respectively whereas NE-AGO-C and NE-AGO-D contained 5% and 10% Tween 80, respectively and NE-AGO-E and NE-AGO-F contained 5% and 10% Triton X-100, respectively. An emulsion inversion point method (EIP) was used to prepare the pre-emulsions. Briefly, the water phase was added to the oil phase under stirring at 50°C for 5 min before subjecting the mixture to high speed stirring using an Ultra-Turrax T25 (Janke and Kunkel GmbH, Germany) at 16,000 rpm for 5 min. The pre-emulsions obtained (20 g) were then passed through a high pressure homogenizer (HPH) (Avestin Inc., Canada) using various homogenization cycles (1, 3, 5, 7, 10, 15, and 20 cycles) with approximately 1–2 min/cycle and pressures of 5,000 and 10,000 psi at room temperature. The experiments were performed at ambient temperature in triplicate.

### Droplet size and zeta potential

The droplet size and size distribution (expressed as polydispersity index, PdI) as well as the zeta potential (ZP) of SMEDDS-AGO and NE-AGO were measured using photon correlation spectroscopy (Zetasizer Nano ZS, Malvern Instruments Ltd., UK). The size measurements were carried out at a fixed angle of 173° at 25°C. All formulations were diluted with distilled water at a ratio of 1:100 (v/v) before measurement. All measurements were carried out in triplicate.

### Conductivity study

The electrical conductivity of SMEDDS-AGO and NE-AGO was measured using a conductivity meter EX-20 (Horiba, Japan). The formulations were diluted in distilled water at a ratio of 1:100 (v/v) before measuring. The conductivity meter was calibrated using a standard solution of 1413 μS/cm (Eutech Instruments) before testing. The experiments were performed at 25°C by dipping the electrode into the test sample until equilibrium was reached and the readings became stable. All measurements were carried out in triplicate.

### Stability study

All SMEDDS-AGO, and NE-AGO formulations were kept at three storage temperatures, 4°C, 20°C, and 40°C, for one month. Changes in optical appearance such as phase separation, color changes, and turbidity were observed visually. The droplet size of the formulations was determined after dispersion the stored samples in distilled water (1:100 v/v) and compared with that of freshly prepared samples investigated in the same manner.

The selected SMEDDS-AGO and NE-AGO kept at different storage conditions (4°C, 20°C, and 40°C) were analyzed for chemical changes using gas chromatography in comparison with AGO. For the SMEDDS-AGO formulations, hexane was added to all samples to get 1:100 dilutions. The diluted samples were mixed using a vortex mixer for 5 min and centrifuged at 13,000 rpm for 1 min. One μL of the supernatant was injected into an Agilent 6890 gas chromatograph equipped with a fused silica capillary column (HP5; 30.0 m × 0.25 mm i.d. × 0.25 μm film thickness) from Agilent Technologies Inc, USA, using flame ionization for detection. For the NE-AGO formulations, all samples were firstly diluted with hexane at a ratio of 1:1 (v/v). Subsequently, the hexane layer was separated and further diluted 100 fold with pure hexane. One μL of the diluted sample was injected into the Agilent 6890 gas chromatograph using the same conditions as described above. All experiments were performed in triplicate.

### Fish and culture condition

Juvenile *Cyprinus carpio* or koi, with an average weight and length of 9.6 ± 0.1 g and 10.9 ± 0.2 cm respectively, were purchased from a local ornamental fish shop in Thailand, and used in the anesthetic study. The fish were maintained in continuously aerated tanks (500 L) with controlled dechlorinated tap water (temperature 25°C; pH 7.4; total hardness 110 ppm; alkalinity 90 ppm; total ammonia nitrogen and nitrite were negative) for 2–4 weeks before being used in the anesthetic study. 50% of fresh water was changed daily. The fish were fed twice daily with a commercial dry feed (INTEQC Feed, Thailand) and held in natural light conditions. All experimental methods were approved by the Animal Care Committee of the Faculty of Veterinary Medicine, Chiang Mai University (FVM-ACUC no. R3/2555).

### Anesthetic effects

Experiments were conducted in glass aquaria (100 × 100 × 150 mm), containing 1 L of dechlorinated tap water. EtOH-AGO, 1:10 v/v dilution in absolute ethanol, was used as a positive control. The selected SMEDDS-AGO and NE-AGO at various concentrations (100, 200, 300, and 400 mg/L) of AGO and EtOH-AGO were added to the aquaria. The fish were divided into 12 groups of 20 individual fish for each treatment and concentration (*n* = 20). The fish were selected randomly and used only once. The criteria to determine the anesthetic effects were based on the fish behavioral responses [23]. The surgical stage where the fish reaches stage 3 plane 2 is the most important for evaluating the efficacy of the anesthetics because at this stage the fish stops any swimming activity, shows loss of equilibrium and loss of responsiveness and is thus easy to handle in routine operations, such as netting, weighing, sorting, operations, vaccinations, and transportation. To evaluate the time required for the induction of fish anesthesia into stage 3 plane 2, the fish were individually anesthetized by each formulation with various AGO concentrations. Furthermore, the anesthetic behavioral changes such as hyperactivity (fish exciting and increased swimming activity), jumping (fish jumping out of water) and piping (fish piping for air near water surface) were also recorded by clinical observation. Each fish was used only once. The maximum observation time was 20 min. When the fish reached the surgical anesthesia stage, they were immediately transferred to anesthetic-free aquaria to measure the recovery time. At the end of the experiment period, the survived fish were maintained in laboratory until they were in healthy condition with no biohazard contamination and then were donated to the aquarist as pet fish.

### Statistical analysis

The data are presented as means ± S.E.M. Kolmogorov-Smirnov’s test was used to evaluate normality of the data distribution. Data was then analyzed by an independent t-test or a one-way ANOVA followed by Tukey’s post-hoc test. Statistical significance was considered at *p*-values < 0.05.

## Results and discussion

### Chemical analysis of AGO

The yield of AGO from the extraction of the fresh rhizomes of *A*. *galanga* was 0.10% v/w. The AGO obtained was a clear, pale yellow liquid. Twenty-six compounds representing 91.42% of the total composition obtained from this oil were identified by GC-MS analysis (Table 1). The main constituents of the extract were oxygenated monoterpenes (1,8-cineole, 37.43%) and phenylpropanoids (4-allylphenyl acetate, 25.97%), which is in agreement with previous reports [24, 25]. Slight differences to the reported chemical compositions of AGO [26–28] are caused by geographic, physiological and genetic variations of the plant material [29].

**Table 1:** Chemical composition of AGO.

Retention time (min)	Compound	Amount (%)*
4.08	α-Pinene	4.53
4.39	Camphene	0.19
4.93	Sabinene	0.32
5.03	β-Pinene	3.27
5.33	β-Myrcene	0.79
6.06	α-Terpipene	0.40
6.60	1,8-Cineole	37.43
7.02	1,3,6-Octatriene	0.03
7.35	γ-Terpinene	0.66
8.32	α-Terpinolene	0.33
11.23	Borneol	0.04
11.32	α Terpineol	2.41
11.73	Terpinen-4-ol	3.24
12.65	2-Butenal	0.22
15.23	Chavicol	1.50
18.93	4-Allylphenyl acetate	25.97
19.23	Eugenol	0.14
20.25	Geranyl acetate	0.77
21.43	Methyl eugenol	5.07
22.79	β-Selinene	0.25
23.08	β-Farnesene	0.15
23.90	Germacrene-D	0.24
25.10	β-Bisaboloene	1.10
25.66	β-Sesquiphellandrene	0.78
26.00	Eugenyl acetate	1.46
30.08	δ-Cadinene	0.13
Total		91.42

* Listed amounts are the percentage of each compound calculated based on the total area of all peaks obtained from the oil.

### Preparation of SMEDDS-AGO

**Fig 1:**
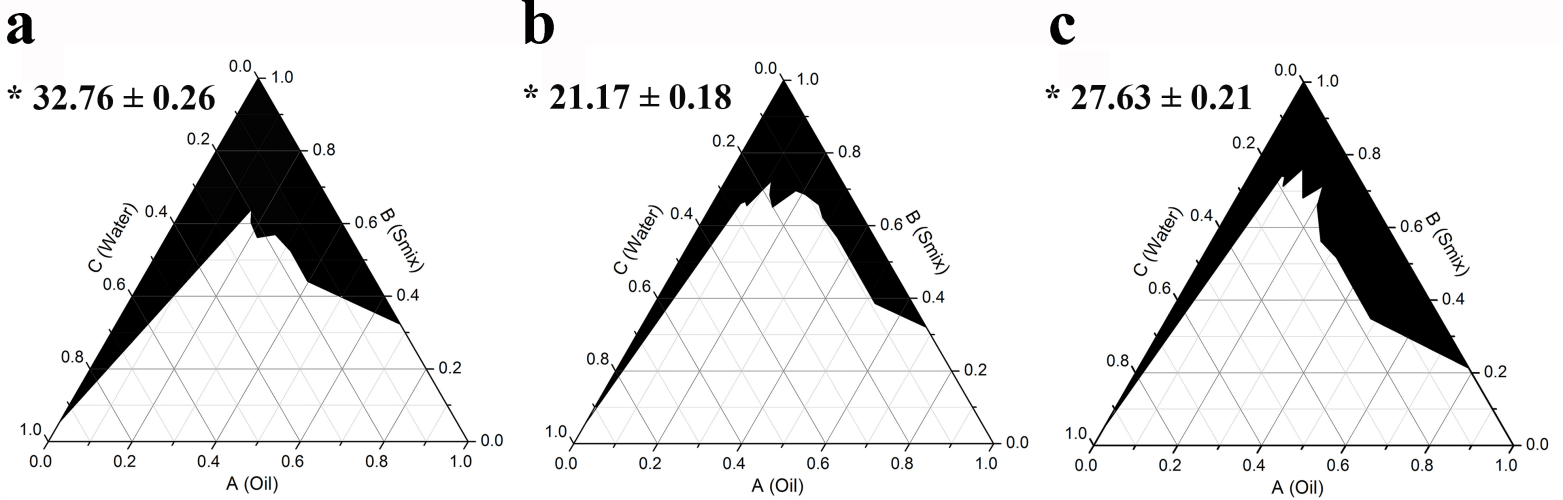
**Pseudoternary phase diagrams of AGO containing Tween 20 (a), Tween 80 (b), and Triton X-100 (c).** The black area represents the monophasic area. Asterisks (*) indicate the percentage of the monophasic area (means ± S.E.M.).

Pseudoternary phase diagrams were constructed to identify the desired SMEDDS regions and to optimize the ratio of oil (AGO), surfactant, and cosurfactant [30]. Initially, the effect of surfactant without cosurfactant on creating a monophasic area in the phase diagram was investigated. It was found that the areas of monophasic transparent formulations using Tween 20, Tween 80, and Triton X-100 were 32.8 ± 0.3, 21.2 ± 0.2, and 27.6 ± 0.2 percent of the total phase diagram, respectively (Fig 1). Thus the results indicate that among the three nonionic surfactants used, Tween 20 created the largest monophasic area in the phase diagrams.

**Fig 2:**
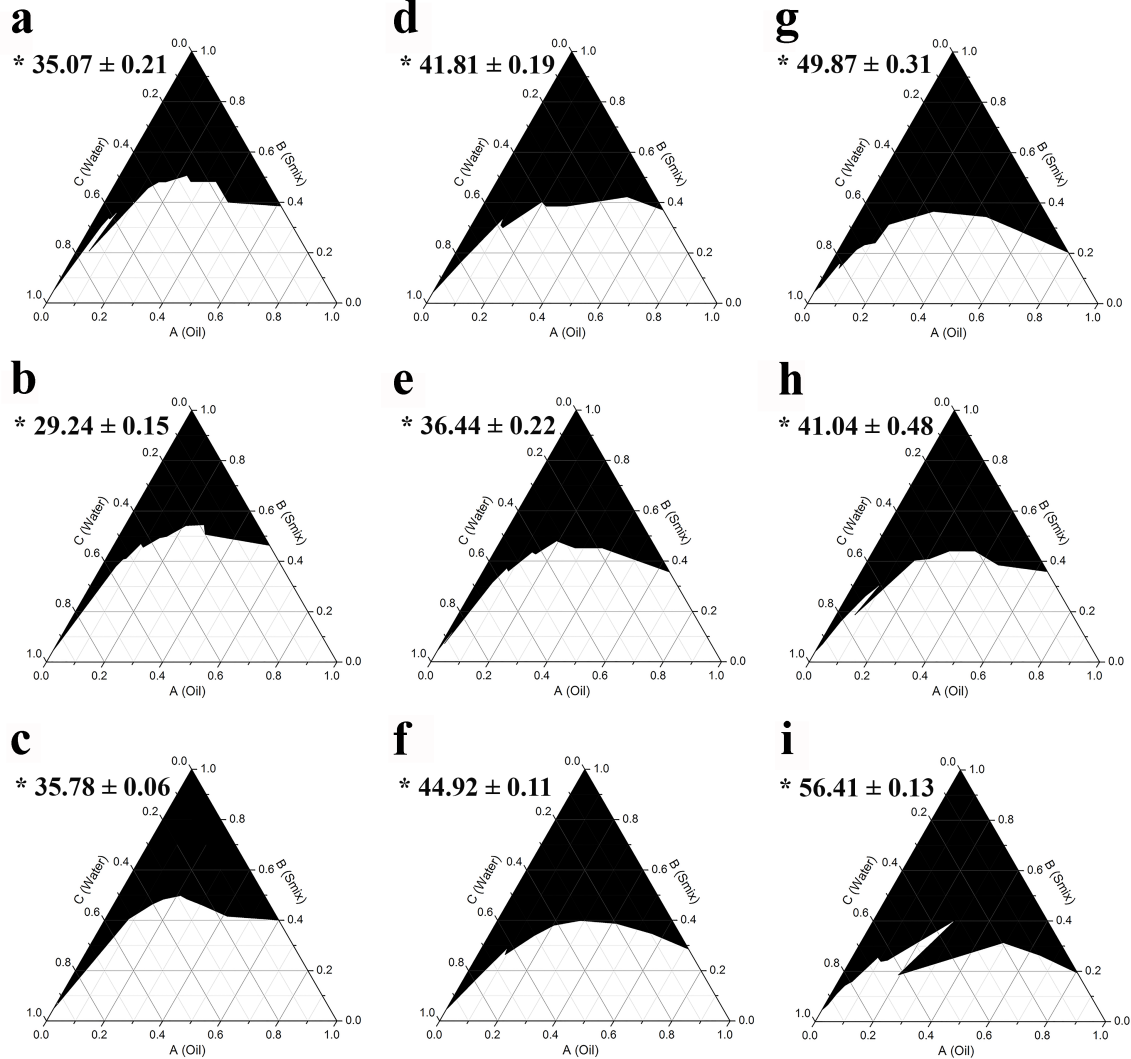
**Pseudoternary phase diagrams of AGO using surfactant:ethanol at a weight ratio of 1:1 (a, d, g), 1:2 (b, e, h), and 2:1 (c, f, i) for Tween 20 (a, b, c), Tween 80 (d, e, f), and Triton X-100 (g, h, i).** The black area represents the monophasic area. Asterisks (*) indicate the percentage of the monophasic area (means ± S.E.M.).

**Fig 3:**
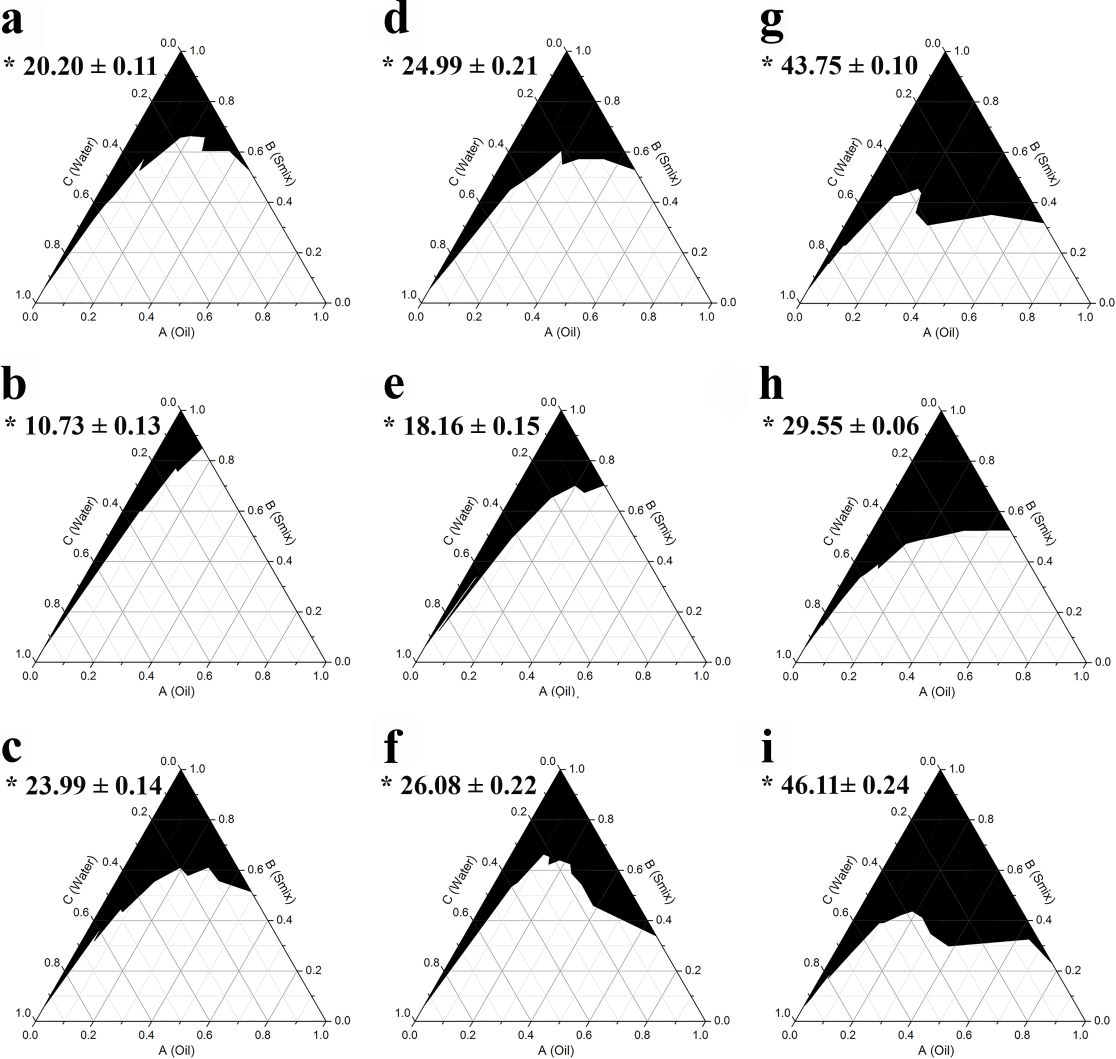
**Pseudoternary phase diagrams of AGO using surfactant:propylene glycol at a weight ratio of 1:1 (a, d, g), 1:2 (b, e, h), and 2:1 (c, f, i) for Tween 20 (a, b, c), Tween 80 (d, e, f), and Triton X-100 (g, h, i).** The black area represents the monophasic area. Asterisks (*) indicate the percentage of the monophasic area (means ± S.E.M.).

**Fig 4:**
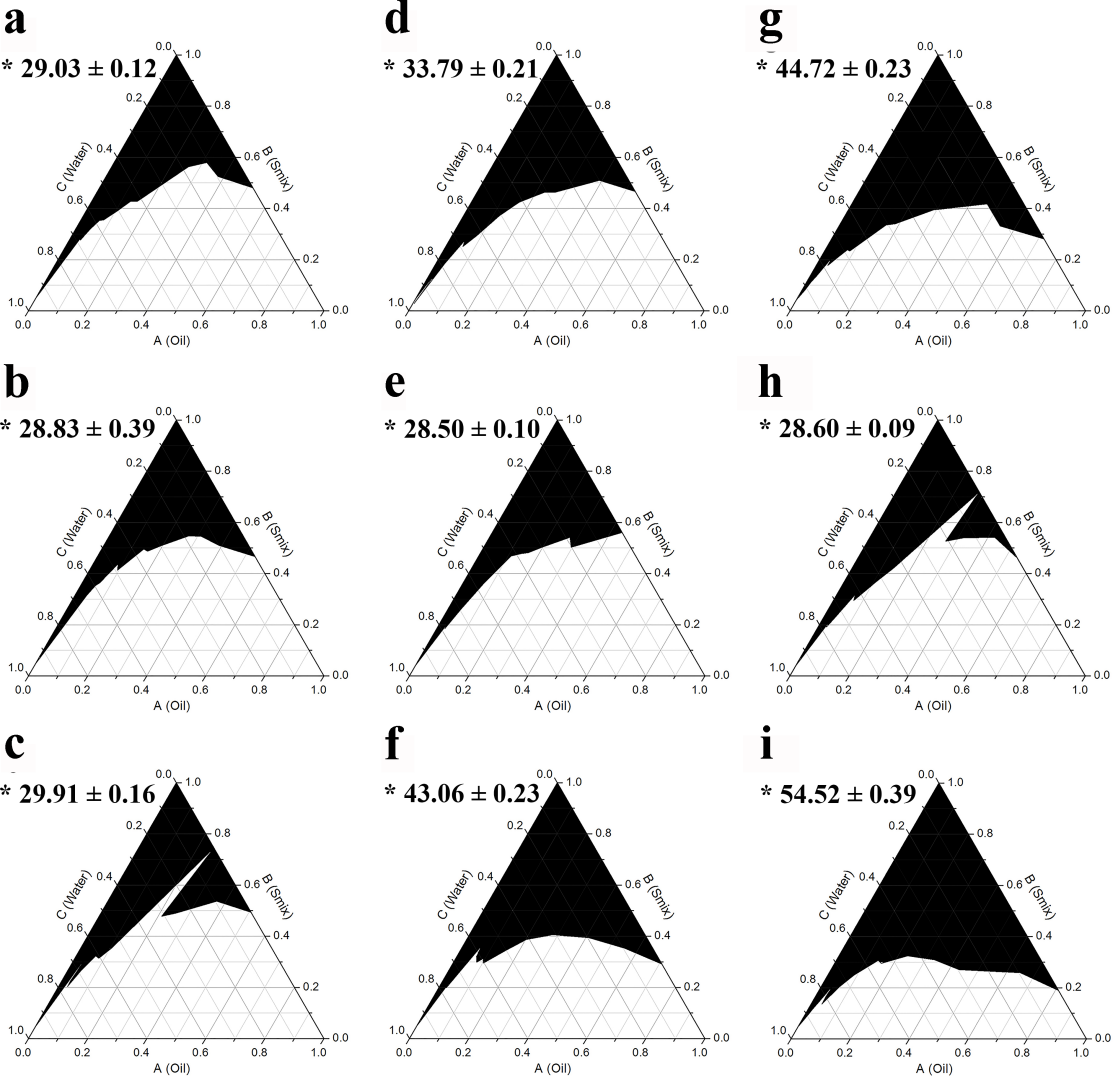
**Pseudoternary phase diagrams of AGO using surfactant:isopropanol at a weight ratio of 1:1 (a, d, g), 1:2 (b, e, h), and 2:1 (c, f, i) for Tween 20 (a, b, c), Tween 80 (d, e, f), and Triton X-100 (g, h, i).** The black area represents the monophasic area. Asterisks (*) indicate the percentage of the monophasic area (means ± S.E.M.).

The use of a single surfactant without cosurfactant is usually not sufficient to reduce the interfacial tension between oil and water to form transparent monophasic systems, at least not in a substantial area of the phase diagram [31, 32]. Thus, the addition of a cosurfactant or cosolvent is generally required. To investigate the effect of cosurfactant on supporting the surfactant to create a monophasic area in the phase diagram, three alcohols; ethanol, propylene glycol, and isopropanol, were used in combination with the three different surfactants at a weight ratio of 1:1, 1:2, and 2:1 (termed Smix). It was found that addition of ethanol, which is more hydrophilic than the other two cosurfactants, resulted in the formation of the largest monophasic area in the phase diagrams as shown in Figs 2–4, respectively. After mixing with the cosurfactants, Triton X-100 showed the highest potential to result in a monophasic area in the phase diagrams, followed closely by Tween 80, whereas the use of Tween 20 resulted in the smallest monophasic area. The results also demonstrated that among the three Smix ratios used (2:1, 1:1, and 1:2), the 2:1 mix was the most effective in creating a large monophasic area.

### Characterization of SMEDDS-AGO

From the obtained pseudoternary phase diagrams, it was found that SMEDDS-AGO composed of 20% of AGO and 80% of Smix (surfactant:ethanol = 2:1) constituted suitable formulations for further characterization. Although using Triton X-100 with ethanol at a weight ratio of 2:1 yielded the largest monophasic area in the phase diagrams, Tween 80 was chosen instead of Triton X-100 because it is less irritating and less toxic than Triton X-100 [33]. Thus, SMEDDS-AGO composed of 20% of AGO and 80% of Smix (Tween 80:ethanol = 2:1) was selected for further characterization. The visual appearance of this SMEDDS-AGO formulation was that of a clear, pale yellowish solution. After 100-fold water dilution, it was found that the average droplet size was 82 ± 0.5 nm with a PdI of 0.38 ± 0.02. The high PdI indicated a fairly broad size distribution in the formulation. Zeta potential, and conductivity of this formulation were –10.40 ± 0.20 mV and 297.7 ± 1.76 μS/cm, respectively. The zeta potential is one of the factors that influences the physical stability of the dispersed droplets, with high positive or negative values (higher than +30 mV or –30 mV, respectively) usually leading to higher physical stability [34]. In this study, diluted SMEDDS showed zeta potential values of less than –30 mV due to the use of a nonionic surfactant. The physical stability of the dispersed droplets of the diluted SMEDDS was however, still high due to steric stabilization of the nonionic surfactant used [35]. The high conductivity value of this formulation confirmed its expected O/W phase behavior.

### Preparation and characterization of NE-AGO

NE formulations are emulsions with a small droplet size in the nanometer range of about 20–200 nm [36]. As can be seen in Table 2, the employed surfactants resulted in different droplet sizes for the various NE-AGO formulations. Generally, an increase in surfactant concentrations led to a decrease in droplet size. After using HPH at a pressure of 10,000 psi for 7 cycles, the largest droplet size (130 ± 0.9 nm) was obtained from NE-AGO-E using 5% of Triton X-100 as a surfactant while NE-AGO-F, using 10% Triton X-100, gave the smallest droplet size (33 ± 0.3 nm).

**Table 2:** Characterization of NE-AGO.

NE-AGO	Size (nm)	PdI	Zeta potential (mV)	Conductivity (μS/cm)
NE-AGO-A	63 ± 1.6	0.38 ± 0.01	–36.3 ± 0.52	511.0 ± 0.33
NE-AGO-B	68 ± 0.9	0.34 ± 0.03	–42.70 ± 0.50	750.7 ± 0.33
NE-AGO-C	104 ± 0.5	0.42 ± 0.00	–35.43 ± 0.38	571.7 ± 0.33
NE-AGO-D	48 ± 1.6	0.43 ± 0.01	–35.07 ± 1.82	551.3 ± 0.33
NE-AGO-E	130 ± 0.9	0.23 ± 0.00	–41.83 ± 0.35	308.0 ± 1.53
NE-AGO-F	33 ± 0.3	0.39 ± 0.01	–31.83 ± 0.35	499.7 ± 0.33

Data are presented as means ± S.E.M.

**Fig 5:**
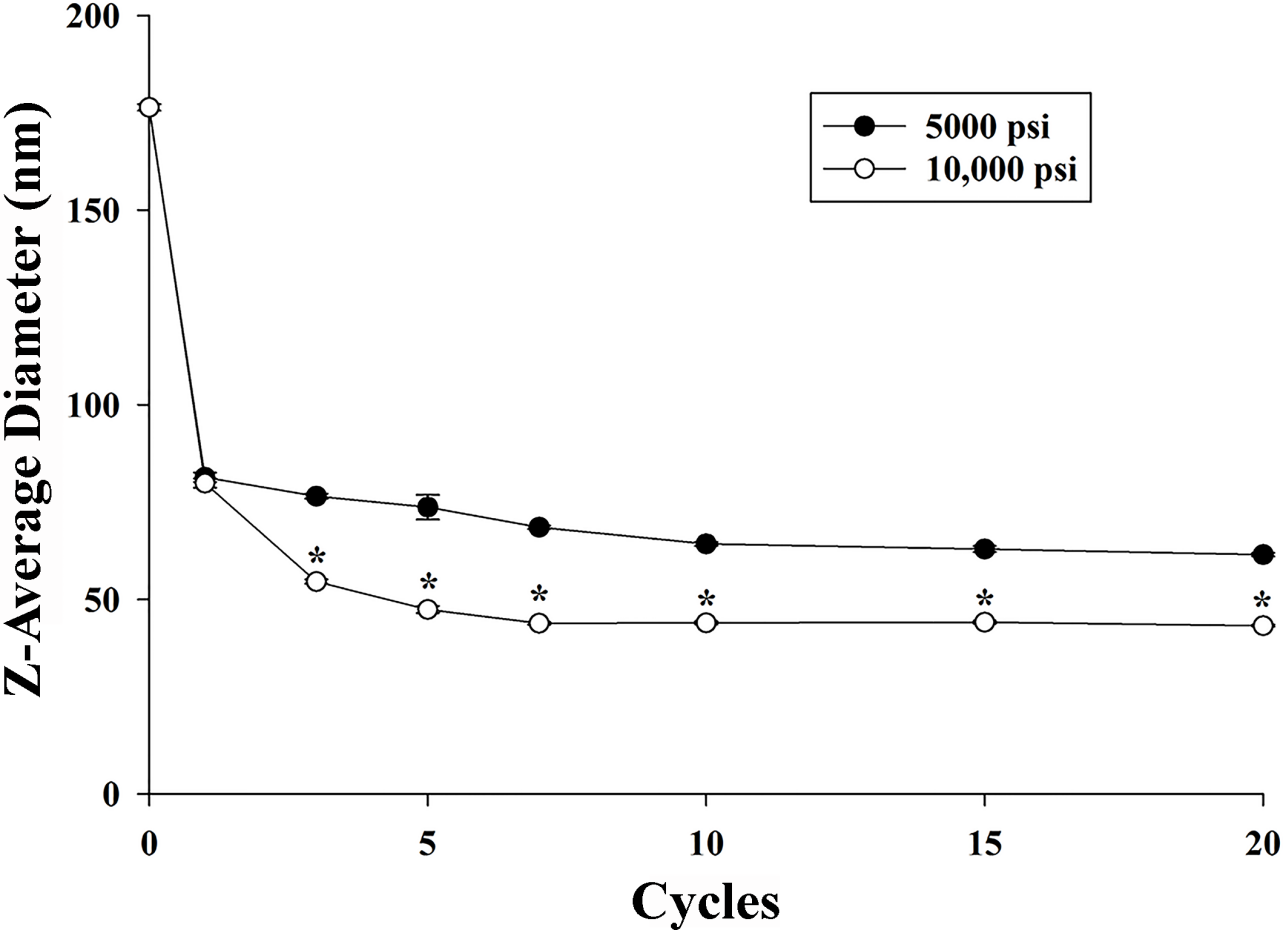
Effect of pressure and number of homogenization cycles on droplet size of the best NE-AGO formulation (NE-AGO-D). Data are presented as means ± S.E.M. (*p* <0.05). Asterisks (*) indicate a significant difference between the obtained NE-AGO-D after using a pressure of 5,000 and 10,000 psi (*p* <0.05). The dataset is available in S1 Dataset.

HPH generated intense disruptive forces, resulting in a decreased droplet radius [37]. The droplet size thus decreased by increasing the HPH pressure and the number of HPH cycles (Fig 5) for NE-AGO-D. The largest decrease of the mean droplet size occurred when the pre-emulsions were passed through the HPH (at all pressures) after the first cycle. In addition, the droplet size of the NE-AGO continued to decrease by increasing the number of HPH cycles, until 7–10 cycles, whereafter no further changes in droplet size were measured.

The six formulations of NE-AGO showed a white bluish color or had a translucent, bluish appearances when 10% of Triton X-100 was used as a surfactant. As shown in Table 2, the smallest droplet size (33 ± 0.3 nm) was obtained from NE-AGO-F while NE-AGO-E had the largest mean droplet size (130 ± 0.9 nm). The PdI values of the NE-AGO formulations were approximately 0.4, except for the NE-AGO composed of 5% Triton X-100 where the PdI was about 0.3. Surprisingly, the zeta potential of all NE-AGO formulations were lower than –30 mV. Since non-ionic surfactants were used, the negative zeta potentials were considered to be due to the spontaneous adsorption of common hydroxyl ions in the aqueous system at the o/w interface [38]. The conductivity of all formulations was found to be higher than 100 μS/cm, which, as expected, confirms the O/W nature of the formed NE in the present study.

### Stability study

The stability results of the selected SMEDDS-AGO is shown in Table 3. The SMEDDS-AGO showed no phase separation after one month of storage. NE-AGO-A, NE-AGO-B, NE-AGO-E, and NE-AGO-F showed phase separation, except NE-AGO-C and NE-AGO-D which were formulated using Tween 80. These two formulations were further characterized and the results are shown in Table 4. NE-AGO-D which contained 10% Tween 80 showed a mean droplet size smaller than the NE-AGO-C that contained 5% Tween 80 at all storage temperatures. Stored for one month, the mean droplet size of NE-AGO-D increased from 48 ± 1.6 nm to 78 ± 2.3 nm at 4°C and to 116 ± 1.2 nm at 40°C. However, the mean droplet size of the formulation stored at 20°C changed only slightly from 48 ± 1.6 nm to 66.1 ± 2.4 nm within one month of storage. Due to its small droplet size, NE-AGO-D was selected for further investigation of chemical stability.

**Table 3:** Characterization of SMEDDS-AGO kept at different temperature for 1 month.

Temperature (°C)	Size (nm)	PdI	Zeta potential (mV)	Conductivity (μS/cm)
4	142 ± 5.3	0.47 ± 0.08	–12.43 ± 1.01	311.0 ± 0.60
20	92 ± 0.8	0.47 ± 0.02	–8.39 ± 0.51	297.1 ± 2.38
40	106 ± 1.5	0.44 ± 0.03	–9.51 ± 0.44	291.13 ± 1.44

Data are presented as means ± S.E.M.

**Table 4:** Characterization of NE-AGO kept at different temperature for 1 month.

NE-AGO^a^	Temperature (°C)	Size (nm)	PdI	Zeta potential (mV)	Conductivity (μS/cm)
NE-AGO-C	4	204 ± 1.7	0.55 ± 0.03	–38.83 ± 0.92	308.3 ± 2.33
	20	144 ± 1.0	0.48 ± 0.02	–21.10 ± 1.14	384.0 ± 1.53
	40	110 ± 1.5	0.46 ± 0.01	–13.30 ± 0.44	484.3 ± 2.19
NE-AGO-D	4	78 ± 2.3	0.50 ± 0.02	–25.50 ± 1.15	321.0 ± 1.15
	20	66 ± 2.4	0.36 ± 0.04	–27.51 ± 0.45	356.0 ± 3.21
	40	116 ± 1.2	0.29 ± 0.01	–22.37 ± 0.82	436.3 ± 2.67

Data are presented as means ± S.E.M.

^a^ = NE-AGO-A, NE-AGO-B, NE-AGO-E, and NE-AGO-F were not determined due to phase separation.

For SMEDDS-AGO, during one month of storage at 20°C, the droplet size of the ME formulation obtained from 1:100 v/v aqueous dilutions before size measurement did not change. On the other hand, the ME formulations obtained from the SMEDDS-AGO stored at 4°C and 40°C showed an increase in the mean droplet size compared to freshly prepared samples (82 ± 0.5 nm) to 142 ± 5.3 nm and 106 ± 1.5 nm, respectively. The storage temperature and time had no influence on the zeta potential of both SMEDDS-AGO and NE-AGO.

**Fig 6:**
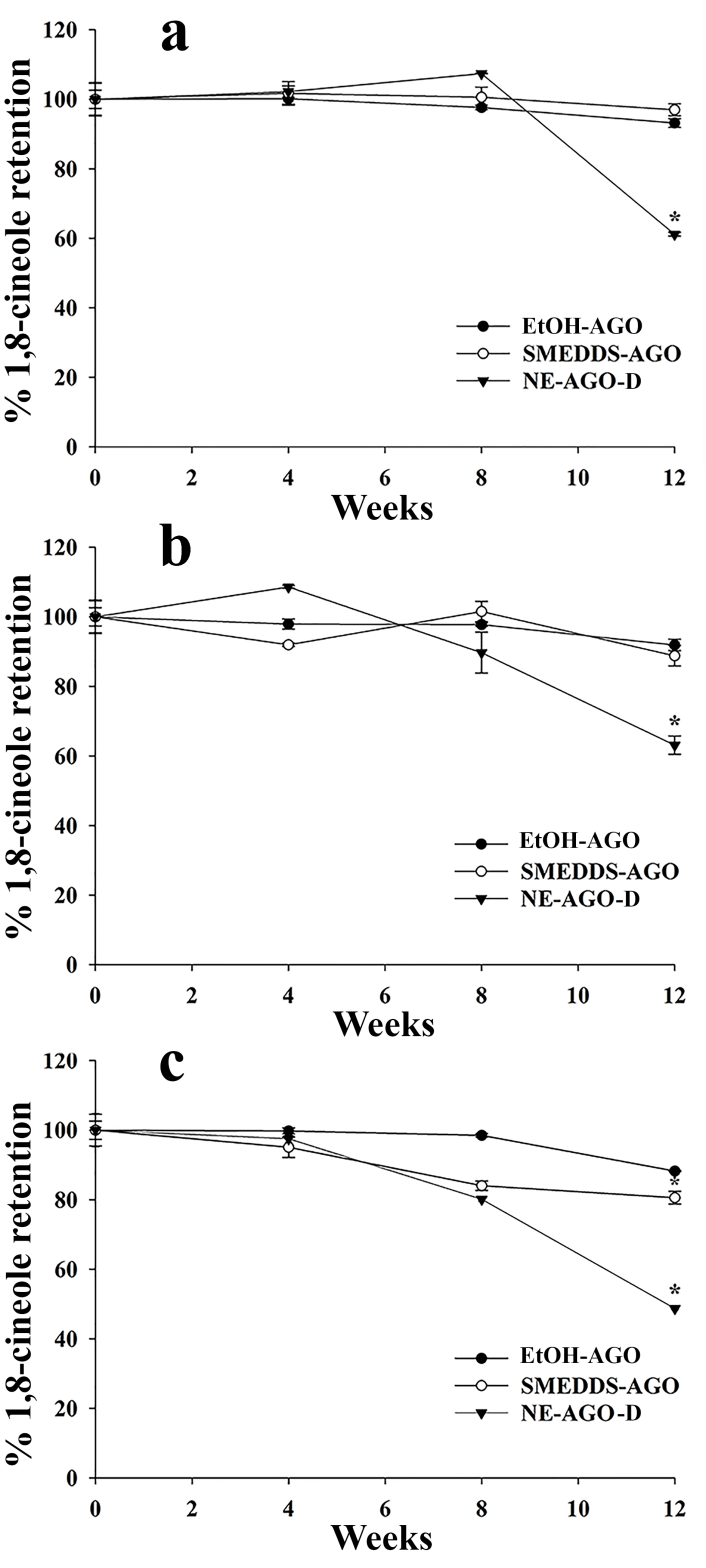
**Chemical stability of 1,8-cineole in SMEDDS-AGO, NE-AGO-D, and AGO at 4°C (a), 20°C (b), and 40°C (c).** Data are presented as means ± S.E.M. Asterisks (*) indicate significant difference between AGO and the developed formulations (SMEDDS-AGO and NE-AGO-D) (*p* <0.05). The dataset is available in S2 Dataset.

**Fig 7:**
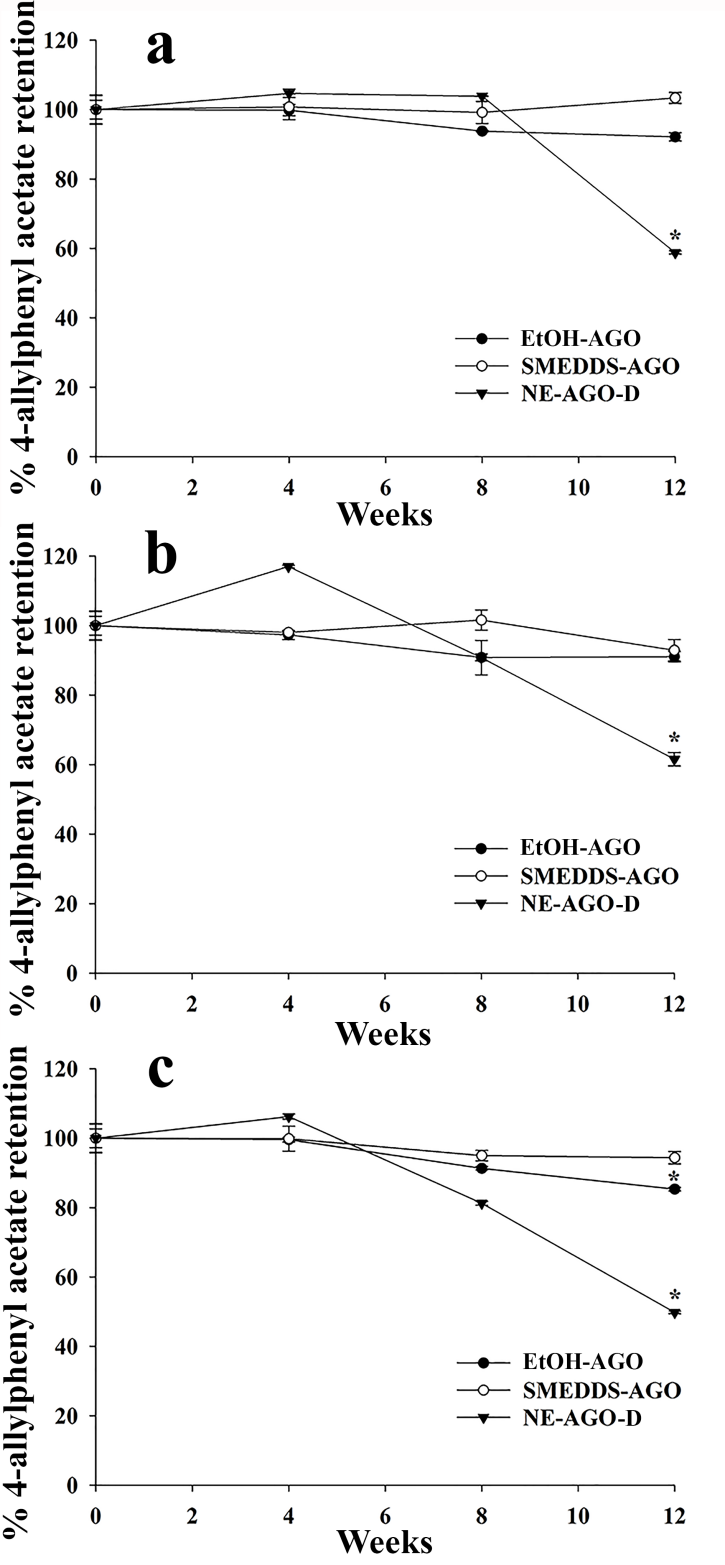
**Chemical stability of 4-allylphenyl acetate in SMEDDS-AGO, NE-AGO-D, and AGO at 4°C (a), 20°C (b), and 40°C (c).** Data are presented as means ± S.E.M. Asterisks (*) indicate significant difference between AGO and the developed formulations (SMEDDS-AGO and NE-AGO-D) (*p* <0.05). The dataset is available in S3 Dataset.

After storage of AGO, SMEDDS-AGO, and NE-AGO-D at 4°C, 20°C and 40°C for 12 weeks, the content of 1,8-cineole and 4-allylphenyl acetate (the main compounds in AGO) was found to have decreased (Figs 6 and 7) and 58%, 60%, and 46% of the initial levels of 1,8-cineole and 55%, 57%, and 46% of the initial levels of 4-allylphenyl acetate were retained in NE-AGO-D stored at 4°C, 20°C, and 40°C, respectively. The decrease of 1,8-cineole content of NE-AGO-D can be explained by oxidation [39] whereas the decrease in 4-allylphenyl acetate content of NE-AGO-D can be explained by hydrolytic degradation in the presence of water in the NE formulation [39, 40]. Moreover, degradation of 1,8-cineole has been reported when the compound was entrapped in a microencapsulated formulation [41, 42]. In contrast, both compounds; 1,8-cineole and 4-allylphenyl acetate, in AGO and SMEDDS-AGO only slightly decreased by not more than 10% of the initial levels at 4°C and 20°C during 12 weeks of storage as seen in Figs 6 and 7, respectively. Thus the presence of water in the formulation was the main problem for AGO stability and SMEDDS demonstrated to be the most stable of the studied systems.

### Anesthetic effect

**Fig 8:**
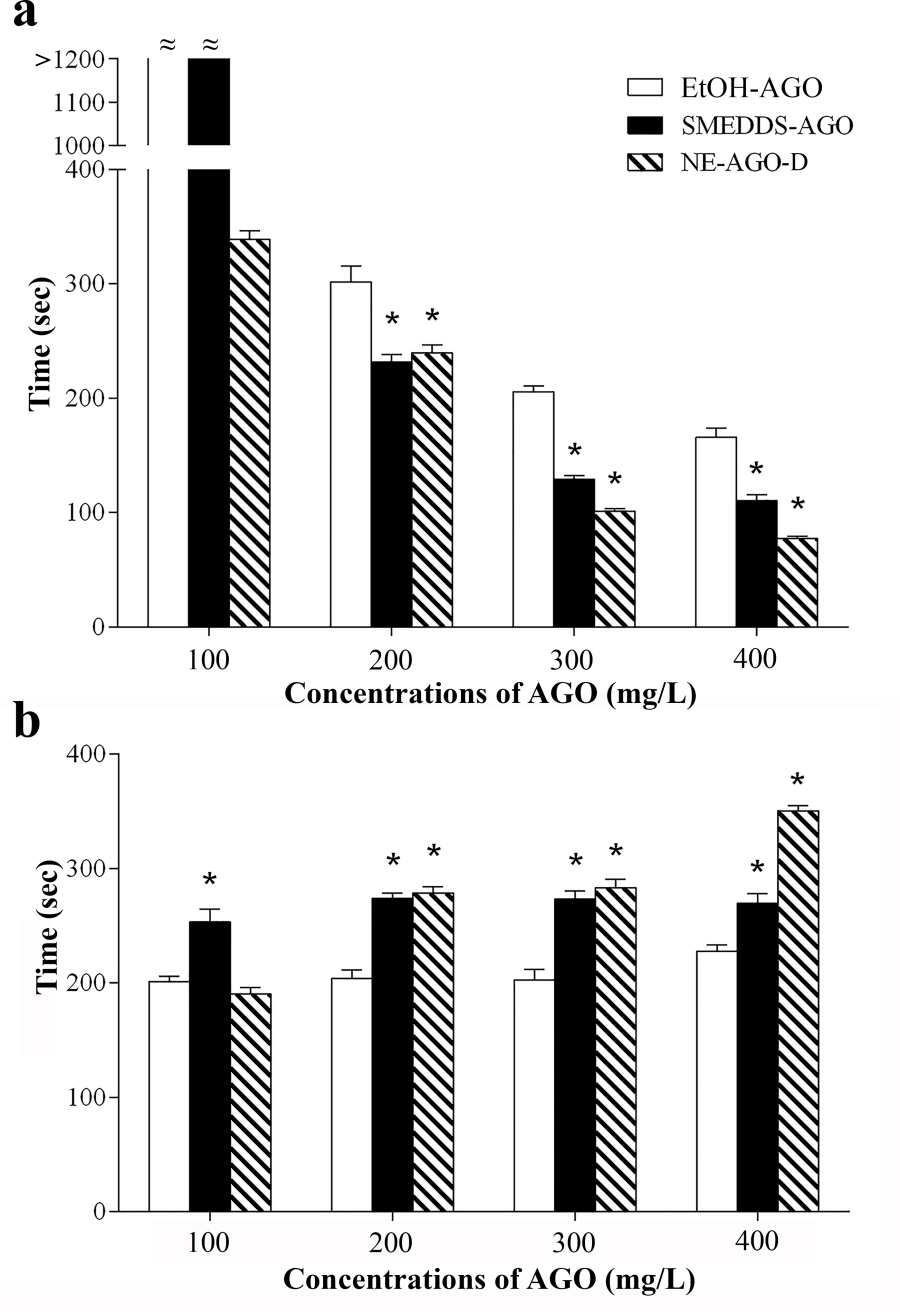
**Induction time (A) and recovery time (B) for *Cyprinus carpio* (*n* = 20) exposed to 100, 200, 300, and 400 mg/L of AGO in SMEDDS-AGO and NE-AGO-D in comparison with EtOH-AGO to reach the surgical anesthesia stage.** Data are presented as means ± S.E.M. Asterisks (*) indicate a significant difference between EtOH-AGO and the developed formulations (SMEDDS-AGO and NE-AGO-D) (*p* <0.05). The dataset is available in S4 Dataset.

Several reports have shown that SMEDDS and NE are systems that can enhance the biological activity of natural compounds [16, 17, 43–46]. Thus, the anesthetic effect of SMEDDS-AGO and NE-AGO-D on *Cyprinus carpio* in comparison with EtOH-AGO was investigated and the results are shown in Fig 8. Both of the developed nanoformulations (SMEDDS-AGO and NE-AGO-D) have a higher anesthetic activity than EtOH-AGO as the induction times of SMEDDS-AGO and NE-AGO-D to reach the surgical anesthesia stage were significantly shorter than those of EtOH-AGO (*p* <0.05) at equal concentrations of AGO (200, 300, and 400 mg/L). However, at a concentration of 100 mg/L, neither EtOH-AGO nor SMEDDS-AGO could induce the fish to stage 3 plane 2 anesthesia which is the surgical stage [23] within an evaluation period of 20 min. Interestingly, at AGO concentration of 100 mg/L using NE-AGO-D, the fish reached the surgical anesthesia stage within approximately 340 sec. At AGO concentrations of 300 and 400 mg/L, NE-AGO-D also showed the highest activity. The fish anesthetized with SMEDDS-AGO or NE-AGO-D at AGO concentrations of 200, 300, and 400 mg/L showed longer recovery times than those anesthetized with EtOH-AGO (*p* <0.05). The recovery times of the fish after SMEDDS-AGO and NE-AGO-D exposure were similar for every AGO concentration tested. After monitoring for 2 weeks, no mortality of the fish occurred after exposure to all selected formulations (SMEDDS-AGO, NE-AGO-D, and EtOH-AGO). Interestingly, it was noted that EtOH-AGO caused unwanted hyperactivity in the fish as seen in Table 5. This side effect did not occur in the fish anesthetized with SMEDDS-AGO and NE-AGO formulations. The results of the present study demonstrated that SMEDDS-AGO and NE-AGO-D enhanced water miscible of AGO and gave higher anesthetic activity with significantly less side effects than EtOH-AGO. The higher anesthetic activity of NE-AGO-D than SMEDDS-AGO was considered to be due to an influence of the droplet size which was significantly smaller for NE-AGO-D than for ME obtained from water dilution of SMEDDS-AGO. No mortality was observed during experimental periods.

**Table 5:** Anesthetic behavioral changes of *Cyprinus carpio* exposed to 100, 200, 300, and 400 mg/L of AGO in SMEDDS-AGO and NE-AGO-D in comparison with EtOH-AGO. The dataset is available in S5 Dataset.

Treatment	Concentration (mg/L)	Anesthetic behavioral changes		
		Hyperactivity	Jumping	Piping
		No. (%)	No. (%)	No. (%)
SMEDDS-AGO	100	0 (0%)	0 (0%)	0 (0%)
	200	0 (0%)	0 (0%)	0 (0%)
	300	0 (0%)	0 (0%)	0 (0%)
	400	0 (0%)	0 (0%)	0 (0%)
NE-AGO-D	100	0 (0%)	0 (0%)	0 (0%)
	200	0 (0%)	0 (0%)	0 (0%)
	300	0 (0%)	0 (0%)	0 (0%)
	400	0 (0%)	0 (0%)	0 (0%)
EtOH-AGO	100	7 (35%)	0 (0%)	0 (0%)
	200	9 (45%)	0 (0%)	0 (0%)
	300	16 (80%)	0 (0%)	0 (0%)
	400	18 (90%)	1 (5%)	3 (15%)

## Conclusion

We have successfully developed SMEDDS-AGO and NE-AGO formulations for enhancing aqueous miscibility of AGO with the avoidance or limitation of alcohol in the formulations. We demonstrate that surfactant and cosurfactant types as well as their composition ratios play an important role for SMEDDS-AGO and NE-AGO formation and stability. We show that water miscibility of AGO can be significantly enhanced by the developed SMEDDS-AGO and NE-AGO formulations. The most suitable formulation for fish anesthesia of SMEDDS-AGO is composed of 20% AGO and 80% surfactant mixture containing 2:1 ratio of Tween 80:ethanol whereas that of NE-AGO is composed of 20% AGO, 10% Tween 80 and 70% water. These nanoformulations showed extremely higher efficacy and significantly less side effect than EtOH-AGO. We conclude that SMEDDS and NE are the promising delivery systems for AGO to enhance water miscibility, decrease the use of alcohol in the formulations, and enhance biological efficacy of AGO.

## Supporting information

S1 Dataset Droplet size of the best NE-AGO formulation (NE-AGO-D) after passing HPH. (XLSX)

S2 Dataset The content of 1,8-cineole in SMEDDS-AGO, NE-AGO-D, and AGO at 4°C, 20°C, and 40°C. (XLSX)

S3 Dataset The content of 4-allylphenyl acetate in SMEDDS-AGO, NE-AGO-D, and AGO at 4°C, 20°C, and 40°C. (XLSX)

S4 Dataset Induction time and recovery time for *Cyprinus carpio* exposed to 100, 200, 300, and 400 mg/L of AGO in SMEDDS-AGO and NE-AGO-D in comparison with EtOH-AGO to reach the surgical anesthesia stage. (XLSX)

S5 Dataset Anesthetic behavioral changes of *Cyprinus carpio* exposed to 100, 200, 300, and 400 mg/L of AGO in SMEDDS-AGO and NE-AGO-D in comparison with EtOH-AGO. (XLSX)
